# Effects of Citrus Peel Hydrolysates on Retrogradation of Wheat Starch

**DOI:** 10.3390/foods10102422

**Published:** 2021-10-13

**Authors:** Ho-Young Park, A-Reum Ryu, Ha Ram Kim, Kwang-Soon Shin, Jung Sun Hong, Hee-Don Choi

**Affiliations:** 1 Research Division of Food Functionality, Korea Food Research Institute, Seongnam-si 55365, Korea; hypark@kfri.re.kr; 2 Research Division of Strategic Food Technology, Korea Food Research Institute, Seongnam-si 55365, Korea; areumryu@kfri.re.kr (A.-R.R.); haram.kim@kfri.re.kr (H.R.K.); jungsunhong@kfri.re.kr (J.S.H.); 3 Department of Food Science and Biotechnology, Kyonggi University, Suwon 16227, Korea; ksshin@kyonggi.ac.kr

**Keywords:** citrus peel, hydrolysates, retrogradation, textural property, wheat starch

## Abstract

Retrogradation is the principal cause for bread staling and, therefore, it has attracted a lot of interest from the food industry. In this study, the inhibitory effect of citrus peel hydrolysates (CPH) on retrogradation of wheat starch (WS) in the presence of sucrose was investigated. The pasting properties showed that further addition of CPH caused a lower setback value than the addition of sucrose alone. Hardness of the gel, retrograded at 4 °C for five days, showed a similar tendency, which was reduced more in CPH addition than WS itself or sucrose addition alone. The low retrogradation enthalpy of the CPH including starch gel also indicated the positive effect of CPH on retarding retrogradation. These results suggested that incorporation of CPH in starch-based foods would be effective for inhibiting retrogradation, preventing the deterioration of the quality of food products.

## 1. Introduction

Starch is the main component of wheat flour, accounting for 80 g/100 g [1]. It is also one of the most common polymers used in the food industry and is widely used to improve the textural properties, viscosity, and stability of starch-based food products [2]. Starch granules are semi-crystalline with a varying crystallinity from 15% to 45% and are composed of different proportions of amylose and amylopectin [3].

Generally, starch is gelatinized before being consumed as food, either during processing or cooking [4]. Starch gelatinization is the irreversible transition of starch granules from a structured to disordered state under high temperature in the presence of water [5]. Gelatinization induces several changes, such as the destruction of the inherent crystalline structure in starch granules [6,7]. Retrogradation involves the destruction of the original granular crystalline structure (gelatinization), and then reconstruction of the novel crystalline structure [8]. The retrogradation of starch mainly depends on the structure of amylose and amylopectin in starch. A higher amylose content results in greater retrogradation [9]. Amylose rearrangement proceeds quickly and begins immediately upon storage. Compared to amylose retrogradation, amylopectin recrystallization proceeds slowly over weeks or even months [9,10].

Retrogradation of starchy foods occurs during food storage. It results in a decrease in water retention capacity and digestibility, and negatively affects the quality of starch-containing food products by destroying the texture and increasing the hardness, opacity, and fragmentation and, therefore, finally affects the storage stability of products [10]. Starch retrogradation is often considered as an undesirable phenomenon because of its major contribution to the staling of bread and other starchy foods, which can cause reduced duration of storage and consumer acceptance and lead to waste [8].

Most starchy foods contain a considerable amount of sugar, especially sucrose, for taste and stability modification, and, therefore, the effect of sucrose on starch properties such as gelatinization and/or retrogradation has been previously investigated [9,11,12,13]. Citrus peels are the primary byproduct from the processing of citrus fruits, whereas they are valuable as a source of dietary fiber [14]. Because they are composed of various types of sugars, their hydrolysates would be used as efficient ingredients for changing retrogradation property of starch. Therefore, the aim of this study was to investigate the effect of citrus peel hydrolysates (CPH) on inhibiting starch retrogradation under the presence of sucrose. We characterized the retrogradation properties of wheat starch (WS) as affected by the addition of sucrose and CPH.

## 2. Materials and Methods

### 2.1. Materials

*Citrus unshiu* were purchased from a local market at Jeju in the Republic of Korea. The pectinases, Pectinex Ultra SP-L and Pectinex Ultra Pulp, were purchased from Novozymes A/S (Bagsvaerd, Denmark). Wheat starch was purchased from Sigma-Aldrich (St. Louis, MO, USA).

### 2.2. Preparation of CPH

Citrus (C. unshiu) peels were dried in an oven at 45 °C. The dried peels were milled using an electric grinder and filtered using a filter sieve to obtain a powdered sample. The citrus peel powder was thoroughly rinsed with distilled water (DW) to remove pigments. Extraction was carried out using two different enzymes for varying durations of enzyme treatment. The resulting solid (solid–liquid ratio of 1:20) was hydrolysed in DW adjusted to pH 4.5 containing 1% Pectinex Ultra Pulp or Pectinex Ultra SP-L and stirred for 3 h or 12 h at 45 °C. The hydrolysates were heated at 100 °C for 10 min to inactivate the enzymes. After enzyme inactivation, the mixture was filtered and centrifuged (1500× *g* for 10 min). The supernatant was precipitated with ethyl alcohol (75%) for 16 h. The mixture was centrifuged (1500× *g* for 10 min) and the supernatant was lyophilised. Extraction yield (%) was calculated as a percentage of the weight of the recovered hydrolysate powder to the weight of the initial citrus peel powder.

### 2.3. Characterization of CPH

#### 2.3.1. Molecular Distribution of CPH

The molecular distribution of CPH was determined using a HPLC (Agilent, Santa Clara, CA, USA) system connected to a Superdex 75 10/300 GL packed column (GE Healthcare, Piscataway, NJ, USA) and a refractive index detector (Agilent). Ammonium formate buffer (pH 5.5, 50 mM) was used for elution at a flow rate of 0.5 mL/min.

#### 2.3.2. The Chemical Composition of CPH

The monosaccharide composition of CPH was analyzed using high-performance anion exchange chromatography coupled with a pulse amperometric detector (HPAEC-PAD). CPH was separated on a CarboPac^TM^ PA1 column (4 × 250 mm with 10 mΜ particle size; Thermo Fisher Scientific, Waltham, MA, USA). The flow rate was a constant 1.0 mL/min and the volume injected was 25 μL. The acidic sugars were eluted with a gradient solvent system using a mobile phase consisting of A (150 mM NaOH) and B (150 mM NaOH with 600 mM NaCH_3_CO_2_) [15]. The neutral sugars were eluted with a gradient solvent system using a mobile phase consisting of A (18 mM NaOH) and B (200 mM NaOH) [16].

### 2.4. Analysis of Starch Retrogradation Adding CPH

The CPH obtained by incubation with Pectinex Ultra Pulp for 12 h was employed to further investigate the effect on the retrogradation property of WS.

#### 2.4.1. Determination of Pasting Properties of CPH with Added Starch

The effect of CPH on the pasting properties of WS was determined using Rapid Visco Analyser (RVA; 4500-TecMaster, Newport Scientific, Warriewood, Australia). The samples were classified into four groups based on the addition of sucrose and CPH. WS slurry was initially prepared by dispersing 3 g (dry basis) WS into 25 g DW (12% (*w*/*v*) suspension) in an RVA aluminium canister, which served as a control. The compositions of the various groups were as follows: Group 2 (WS + sucrose), 0.6 g of sucrose was added to 3 g of WS; Group 3 (WS + sucrose + CPH10) and Group 4 (WS + sucrose + CPH20); 0.6 g of sucrose and 0.3 g or 0.6 g of CPH were added, respectively. The test profile was programmed according to the standard pasting method.

#### 2.4.2. Determination of Textural Properties of CPH with Added Starch Gel

We investigated the effect of CPH on the starch retrogradation using a textural analyzer in texture profile analysis (TPA) mode. The starch pastes that were produced after RVA measurement were cooled to be set as starch gel and then stored at 4 °C for zero, one, three, and five days for retrogradation. The size of the gel used for TPA was 5.0 cm × 2.0 cm (diameter × height). The gels were compressed twice with a cylinder probe with 25 mm diameter at a test speed of 0.5 mm/s. Compression of the starch gel was set to 50% of the original sample height. The textural parameters of hardness were calculated from the TPA curves.

#### 2.4.3. Determination of Thermal Properties of CPH with Added Starch Gel

The starch pastes produced via RVA were stored at 4 °C for five days and were lyophilized. The powered sample and distilled water was weighed into a stainless-steel pan at the ratio of 1:3. The pan was hermetically sealed and then kept at room temperature overnight for moisture equilibrium. Thermal analysis of the retrograded starch gels was performed using a differential scanning colorimeter (DSC; DSC 4000, Perkin Elmer, Waltham, MA, USA). The scan was conducted from 25 °C to 95 °C at a 10 °C/min scanning rate, with an empty pan as a reference. The onset, peak, and end temperatures and enthalpies corresponding to retrogradation were measured.

### 2.5. Statistical Analysis

Data are expressed as mean ± standard deviation (SD), and the samples were analyzed via a one-way analysis of variance using SAS, version 8.1 (SAS Institute, Cary, NC, USA). Differences with a *p* value < 0.05 were considered significant.

## 3. Results and Discussion

### 3.1. Comparison of CPH Extraction Yield

Extraction yields of CPH after treatment with the two enzymes are presented in Table 1. The extraction yields of CPH obtained from Pectinex Ultra Pulp treatment were 18.6% and 22.8% for 3 h and 12 h incubation, respectively, and those from Pectinex Ulta SP-L treatment were 16.7% and 21.8%, respectively. Thus, there was no significant difference between the two enzyme treatments. The extraction yields were increased by 1.1 times along with the increase in the enzyme treatment’s duration. Therefore, it could be concluded that the type of enzyme treatment did not affect CPH yield. However, an increased time of enzyme treatment increased the extraction yield.

### 3.2. Characterization of CPH Composition

**Figure 1:**
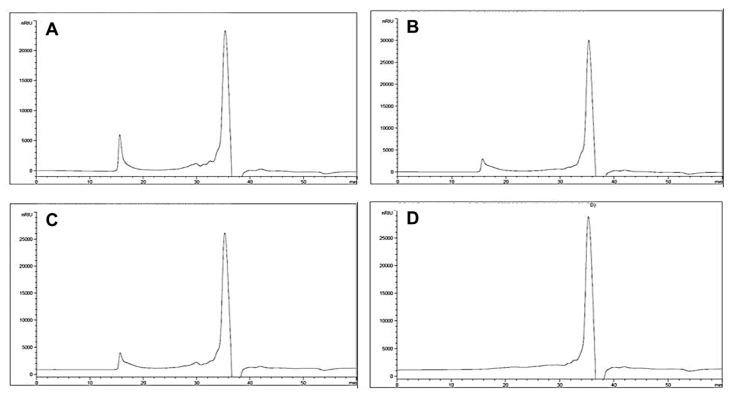
Changes of HPLC chromatogram with different types of enzyme and incubating times using citrus peel hydrolysate (CPH). (**A**), Pectinex Ultra SP-L 3 h; (**B**), Pectinex Ultra SP-L 12 h; (**C**), Pectinex Ultra Pulp 3 h; (**D**), Pectinex Ultra Pulp 12 h.

The molecular distributions of CPH observed via HPLC separation were presented in Figure 1. A single purified peak was revealed in the chromatogram of Pectinex Ultra Pulp treated CPH for 12 h, while others showed bimodal distribution, implying incomplete and uneven hydrolyzation. The major monosaccharide was found to be arabinose (29.2–30.4%,), followed by glucose (24.8–26.8%) and galacturonic acid (21.4–24.9%), constituting 78–80% of the total monosaccharide content (Table 1). The monosaccharide compositions of citrus peels, such as lemon and grapefruit, reported in a previous study, were consistent with our results [14]. There was no significant difference in the composition of CPH from different enzyme treatments and treatment duration. Among the four samples, CPH produced by Pectinex Ultra Pulp treatment for 12 h, which showed rather unimodal distribution and the highest yield, was selected for further evaluation.

### 3.3. The Effect of CPH on Starch Retrogradation

#### 3.3.1. The Effect of CPH on the Pasting Properties of Starch

**Figure 2:**
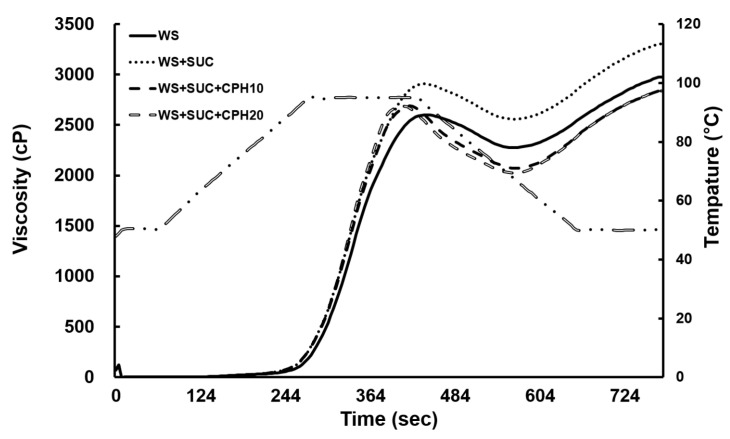
Pasting curves of wheat starch with added sucrose and citrus peel hydrolysates. WS, wheat starch; SUC, sucrose; CPH10, citrus peel hydrolysate 10%; CPH20, citrus peel hydrolysate 20%.

We evaluated the effect of CPH on the pasting properties of WS by RVA, and the results are presented in Figure 2 and Table 2. WS slurry was used as a control to compare the pasting properties of mixtures of CPH and sucrose. The addition of sucrose and CPH resulted in the slight increase in the peak viscosity of WS, which corresponded with the previous report [11]. It is known that the swelling of starch granules progresses more slowly in a sucrose solution than in pure water [17]. Moreover, changes in the interactions of starch and water, affected by sucrose and CPH, possibly altered the pasting behavior of WS. It is possible that this ultimately leads to a greater diameter of swollen granules [17], resulting in the changes in paste viscosity and pasting temperature.

Breakdown is associated with the stability of starch paste [18]. The breakdown of WS was 744.0 cP, and increased to the value of 805.3 cP in sucrose-added starch. In the starch with both CPH and sucrose added, this value was lowered depending on CPH concentration, implying that CPH nullifies the effect of sucrose on the stability of WS paste.

Setback viscosity values depend on the difference between trough viscosity and final viscosity, which results from the rearrangements between amylose and amylopectin when the temperature was decreased from 95 °C to 50 °C [18]. Therefore, setback values can predict the degree of starch retrogradation. As shown in Table 2, the setback viscosity value of WS was 1199.7 cP, and the value increased slightly to 1284.7 cP when sucrose was added. However, in the mixtures of CPH and sucrose with WS, the setback viscosity values significantly decreased to 872.7 cP and 823.7 cP at CPH concentrations of 10% and 20%, respectively. Previous study observed the increase in setback viscosity with increasing sucrose content [11], which corresponded with the result from this study. On the contrary, the further addition of CPH successfully decreased the final viscosity and setback compared with those of WS + sucrose and WS. It strongly implied the inhibitory effect of CPH on retrogradation of starch in the presence of sucrose. CPH seemed to function as a diluent of retrogradation, inhibiting the accelerating action of sucrose. Its various sugar composition with different hydrophilic/hydrophobic properties, as presented in Table 1, would contribute to this attribute.

#### 3.3.2. The Effect of CPH on Textural Properties of Starch Gel

**Figure 3:**
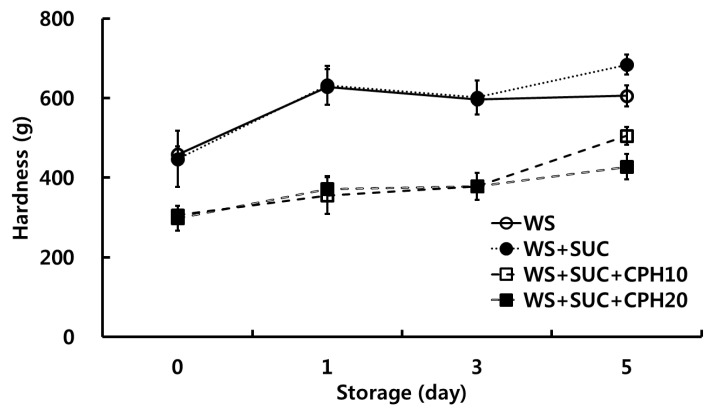
Effects of citrus peel hydrolysate on hardness of wheat starch. WS, wheat starch; SUC, sucrose; CPH10, citrus peel hydrolysate 10%; CPH20, citrus peel hydrolysate 20%.

Starch retrogradation is a major factor in the staling of bread and rice cake, and is thought to affect textural properties, for example, hardness [19]. Therefore, we analyzed the textural properties of retrograded starch gel containing sucrose by TPA in the presence or absence of CPH (Figure 3). The textural parameters of hardness were calculated from the TPA curves, being defined as the maximum force during the first compression [10]. As shown in Figure 3, the hardness of the gel increased during the storage period, showing the highest value at five days of storage. After being stored for five days, the hardness of retrograded WS gel was 605.7 g. The hardness of retrograded WS + sucrose gel was higher (684.0 g) than that of the control. According to the previous studies, the addition of sucrose increased the firmness of the starch gel [13], because sucrose has a considerable hydration ability and induces more associations of starch molecules [20]. This corresponded with the observations from this study. The hardness value of WS gel with sucrose and CPH of 10% and 20% were 505.6 g and 427.5 g, which corresponded with the 73.9% and 62.5% of WS + sucrose gel, respectively. These results suggest that the addition of CPH was effective in retarding starch retrogradation.

#### 3.3.3. The Effect of CPH on the Thermal Properties of Starch Gel

We measured the thermal properties of gel stored at 4 °C for five days to analyze the retrogradation characteristics by DSC (Table 3). Starch molecules in the gelatinized paste reassociate and form an ordered structure, composed of double helices, during retrogradation [8]. Thus, the retrogradation process includes crystallization.

DSC analysis reflects the property of the double helical structure and the hydrogen bonds that stabilize the double helical structure. The melting temperature indicates the structural stability of crystallites of the degree of the order of double helices, where changes in ΔH indicate the energy required for thermal transition of double helices and the resultant crystallites [21].

The onset, peak, and end temperatures of the lyophilized gels were revealed over the temperature range of 54.19–59.54 °C, with no significant difference among samples. However, a difference according to the addition of sugar and CPH was observed in ΔH values, which reflect the different degree of retrogradation. After five days of storage, the ΔH value of WS was 0.354 J/g and that of WS + sucrose gel was 0.367 J/g. The addition of CPH by 10% and 20% resulted in a significant decrease in retrogradation enthalpy of 0.178 J/g and 0.212 J/g, respectively. These values corresponded to 74% and 63% of the ΔH from WS + sucrose gel. A lower ΔH value indicates that a smaller amount of crystalline structure, as well as the double helical structure which originated from the hydrogen bonds regenerated during retrogradation, was formed in starch gels that included CPH. Therefore, compared with WS itself or WS with only sucrose added, retrogradation was retarded when CPH was also included. This result corresponded with RVA pasting properties (especially setback) and gel hardness.

The fundamental principle of starch retrogradation is the conversion of hydrogen bonds between the starch molecule and the water molecule into hydrogen bonds among the starch molecules [8]. Therefore, as described above, when hydrogen bonds in the starch paste are affected by hydrophilic or hydrophobic ingredients, retrogradation behavior can be altered. Unlike mono-/di-/oligo-saccharides, which did not have a positive effect on retarding retrogradation [9,11,17,18], CPH could successfully inhibit the retrogradation of starch as proved by multiple methods, such as pasting property, gel texture, and DSC analysis.

## 4. Conclusions

CPH was prepared by the enzymatic hydrolysis of citrus peel, and its effects on the retrogradation of starch paste, including sucrose, were examined. The CPH was composed of various monosaccharides where arabinose, glucose, and galacturonic acid were dominant (78–80%). Though sucrose addition did not have a positive effect on retarding retrogradation, further addition of CPH significantly inhibited the retrogradation, as supported by the lower setback value (RVA), lower retrograded gel hardness, and lower retrogradation enthalpy. This research showed that adding CPH to starch-based foods, particularly sucrose-containing products such as cake and sweet dough bread, is effective for inhibiting retrogradation. Therefore, CPH could be utilized as a novel food ingredient in the form of an anti-staling agent to stabilize food quality for a longer storage time.

## Figures and Tables

**Table 1:** Extraction yield and monosaccharide composition of citrus peel hydrolysates treated with different types of enzyme and incubation times.

	Pectinex Ultra SP-L		Pectinex Ultra Pulp	
	Treatment Time (h)		Treatment Time (h)	
	3	12	3	12
Extraction yield (%)	16.7	21.8	18.6	22.8
Monosaccharides content (mg/g)				
Fucose	4.0 ± 0.1	3.8 ± 0.1	3.4 ± 0.1	4.0 ± 0.0
Rhamnose	14.6 ± 0.1	12.3 ± 0.1	13.3 ± 0.1	10.6 ± 0.2
Arabinose	137.4 ± 0.6	129.9 ± 0.6	133.3 ± 1.7	132.0 ± 1.3
Galactose	67.0 ± 0.8	60.3 ± 0.1	62.0 ± 0.5	58.8 ± 0.8
Glucose	122.4 ± 1.1	112.8 ± 0.4	108.9 ± 1.2	114.5 ± 1.3
Mannose	7.4 ± 0.0	9.2 ± 0.0	3.7 ± 0.1	8.5 ± 0.3
Xylose	5.7 ± 0.5	7.0 ± 0.1	4.9 ± 0.3	7.3 ± 0.2
Galacturonic acid	97.7 ± 5.0	110.1 ± 4.7	109.5 ± 4.1	105.5 ± 1.8

**Table 2:** Pasting properties of wheat starch with added sucrose and citrus peel hydrolysates.

	Peak Viscosity (cP)	Trough Viscosity (cP)	Breakdown (cP)	Final Viscosity (cP)	Setback (cP)	Pasting Temp. (°C)
WS	2518.3 ± 26.6 ^c^	1774.3 ± 56.6 ^b^	744.0 ± 31.5 ^a^	2974.0 ± 58.6 ^a^	1199.7 ± 5.1 ^b^	93.9 ± 0.6 ^a^
WS + SUC	2824.3 ± 42.0 ^a^	2068.0 ± 25.5 ^a^	805.3 ± 51.5 ^a^	3303.7 ± 71.8 ^a^	1284.7 ± 18.6 ^a^	90.0 ± 0.4 ^b^
WS + SUC + CPH10	2684.3 ± 7.5 ^b^	1965.0 ± 90.7 ^a^	719.3 ± 87.4 ^ab^	2837.7 ± 22.9 ^a^	872.7 ± 71.8 ^c^	90.3 ± 0.9 ^b^
WS + SUC + CPH20	2630.0 ± 46.7 ^b^	2013.0 ± 64.2 ^a^	676.0 ± 49.5 ^b^	2836.7 ± 128.1 ^a^	823.7 ± 76.1 ^c^	90.6 ± 1.4 ^b^

WS, wheat starch; SUC, sucrose; CPH10, citrus peel hydrolysate 10%; CPH20, citrus peel hydrolysate 20%. Different letters indicate significant differences within the column (*p* < 0.05).

**Table 3:** Thermal properties of the wheat starch gel added with sucrose and citrus peel hydrolysates stored for 5 days.

	Onset Temp. (°C)	Peak Temp. (°C)	End Temp. (°C)	Retrogradation Enthalpy (ΔH, J/g)
WS	54.71 ± 0.23 ^ab^	57.37 ± 0.20 ^a^	58.29 ± 0.19 ^b^	0.354 ± 0.009 ^a^
WS + SUC	54.90 ± 0.18 ^a^	56.87 ± 0.49 ^b^	59.54 ± 0.33 ^a^	0.367 ± 0.010 ^a^
WS + SUC + CPH10	54.70 ± 0.11 ^ab^	55.87 ± 0.25 ^b^	57.58 ± 0.21 ^c^	0.178 ± 0.023 ^b^
WS + SUC + CPH20	54.19 ± 0.31 ^b^	56.86 ± 0.57 ^b^	59.05 ± 0.21 ^a^	0.212 ± 0.014 ^b^

WS, wheat starch; SUC, sucrose; CPH10, citrus peel hydrolysate 10%; CPH20, citrus peel hydrolysate 20%. Different letters indicate significant differences within the column (*p* < 0.05).

## Data Availability

Data are contained within the article.
